# Correlation between Bioassay and Protein Misfolding Cyclic Amplification for Variant Creutzfeldt-Jakob Disease Decontamination Studies

**DOI:** 10.1128/mSphere.00649-19

**Published:** 2020-01-29

**Authors:** Maxime Bélondrade, Christelle Jas-Duval, Simon Nicot, Lilian Bruyère-Ostells, Charly Mayran, Laetitia Herzog, Fabienne Reine, Juan Maria Torres, Chantal Fournier-Wirth, Vincent Béringue, Sylvain Lehmann, Daisy Bougard

**Affiliations:** a Pathogenesis and Control of Chronic Infections, Etablissement Français du Sang, INSERM, Université de Montpellier, Montpellier, France; b VIM INRA, Université Paris-Saclay, Jouy-en-Josas, France; c Centro de Investigación en Sanidad Animal, Instituto Nacional de Investigación y Tecnología Agraria y Alimentaria (CISA-INIA), Madrid, Spain; d CHRU de Montpellier and Université de Montpellier, IRMB, INSERM U1183, Laboratoire de Biochimie Protéomique Clinique, Montpellier, France; Colorado State University

**Keywords:** PMCA, bioassay, decontamination, prion, variant Creutzfeldt-Jakob disease

## Abstract

To date, approximately 500 iatrogenic Creutzfeldt-Jakob disease cases have been reported worldwide, most of them resulting from cadaveric dura mater graft and from the administration of prion-contaminated human growth hormone. The unusual resistance of prions to decontamination processes, their large tissue distribution, and the uncertainty about the prevalence of variant Creutzfeldt-Jakob disease (vCJD) in the general population lead to specific recommendations regarding identification of tissue at risk and reprocessing of reusable medical devices, including the use of dedicated treatment for prion inactivation. We previously described an *in vitro* assay, called Surf-PMCA, which allowed us to classify prion decontamination treatments according to their efficacy on vCJD prions by monitoring residual seeding activity (RSA). Here, we used a transgenic mouse line permissive to vCJD prions to study the correlation between the RSA measured *in vitro* and the *in vivo* infectivity. Implantation in mouse brains of prion-contaminated steel wires subjected to different decontamination procedures allows us to demonstrate a good concordance between RSA measured by Surf-PMCA (*in vitro*) and residual infectivity (*in vivo*). These experiments emphasize the strength of the Surf-PMCA method as a rapid and sensitive assay for the evaluation of prion decontamination procedures and also confirm the lack of efficacy of several marketed reagents on vCJD prion decontamination.

**IMPORTANCE** Creutzfeldt-Jakob diseases are neurodegenerative disorders for which transmission linked to medical procedures have been reported in hundreds of patients. As prion diseases, they are characterized by an unusual resistance to conventional decontamination processes. Moreover, their large tissue distribution and the ability of prions to attach to many surfaces raised the risk of transmission in health care facilities. It is therefore of major importance that decontamination procedures applied to medical devices before their reprocessing are thoroughly validated for prion inactivation. We previously described an *in vitro* assay, which allowed us to classify accurately prion decontamination treatments according to their efficacy on variant Creutzfeldt-Jakob disease. The significance of this study is in demonstrating the concordance between previous *in vitro* results and infectivity studies in transgenic mice. Furthermore, commercial reagents currently used in hospitals were tested by both protocols, and we observed that most of them were ineffective on human prions.

## Abstract (precis)

Creutzfeldt-Jakob diseases are neurodegenerative disorders for which transmission linked to medical procedures have been reported in hundreds of patients. As prion diseases, they are characterized by an unusual resistance to conventional decontamination processes. Moreover, their large tissue distribution and the ability of prions to attach to many surfaces raised the risk of transmission in health care facilities. It is therefore of major importance that decontamination procedures applied to medical devices before their reprocessing are thoroughly validated for prion inactivation. We previously described an *in vitro* assay, which allowed us to classify accurately prion decontamination treatments according to their efficacy on variant Creutzfeldt-Jakob disease. The significance of this study is in demonstrating the concordance between previous *in vitro* results and infectivity studies in transgenic mice. Furthermore, commercial reagents currently used in hospitals were tested by both protocols, and we observed that most of them were ineffective on human prions.

## INTRODUCTION

Prion diseases, or transmissible spongiform encephalopathies (TSE), are a group of fatal neurodegenerative disorders affecting mammals, including scrapie in sheep, bovine spongiform encephalopathy in cattle, chronic wasting disease in cervids and Creutzfeldt-Jakob disease (CJD) in humans (1). They are characterized by the posttranslational modification of the host-encoded cellular prion protein (PrP^C^) into an abnormal aggregated isoform (PrP^TSE^) capable of self-propagation through autocatalytic templating activity and of wide accumulation in the central nervous system (2, 3). PrP^TSE^ has also been detected in various tissues and organs of patients with CJD (4–6), including eyes, skin, and blood, which suggests a risk of interindividual transmission by medical procedures (7–10). To date, ∼500 iatrogenic CJD cases have been reported worldwide, most of which result from cadaveric dura mater grafts and from the administration of prion-contaminated human growth hormone (11, 12).

Owing to their aggregative nature, prions are highly resistant to conventional chemical and physical decontamination methods (13, 14). Moreover, prions readily bind to many surfaces, especially stainless steel, rendering decontamination even more challenging (15, 16). The difficulties in giving a definitive diagnosis of CJD antemortem poses a serious threat to the control of infection in health care facilities, and cautionary measures are needed to prevent further iatrogenic transmission using surgical instruments. Consequently, specific preventive recommendations have been proposed in medical practice. Guidelines regarding prion decontamination protocols for reusable surgical instruments and surfaces (1999) and tissue infectivity distribution in TSE (2006 to 2010) were addressed by the WHO (17, 18). These guidelines aimed to identify patients and surgical procedures at risk (e.g., neurosurgery, otorhinolaryngology in cases where there is contact with the olfactory mucosa, ophthalmology in cases where there is contact with the retina or optic nerves) and give specific recommendations regarding the decontamination of nondisposable medical devices (MDs). Recommended methods for prion decontamination include the use of high concentrations of sodium hydroxide or sodium hypochlorite. While less efficient, prion inactivation by autoclaving at 134°C (steam and porous load autoclave) has also been proposed and is now often implemented in hospitals. However, these harsh treatments are not compatible with fragile and expensive MDs contaminated with high-risk tissues, which need to be treated specifically against prions before their reuse. Specific prion decontamination processes have therefore been developed to ensure the reprocessing of fragile MDs, and alternative approaches using alkaline, acidic, enzymatic, or phenolic compounds, hydrogen peroxide and copper mixes, as well as gaseous hydrogen peroxide, have been proposed by several groups (19–24). An important issue concerns the validation of these processes in terms of their prion inactivation and decontamination capacities. Currently, validation of new treatments relies on expensive, time-consuming, and ethically challenging animal bioassay infectivity studies, using nonhuman prion strains associated with steel wires as model carrier (25). In France, prion reduction steps include commercial products endorsed by the French regulatory agency (Agence Nationale de Sécurité du Médicament et des Produits de Santé [ANSM]) after their validation using the well-documented hamster infectivity assays using the 263K prion strain adsorbed and dried on steel wires. Although this methodology simplifies the comparison for decontamination efficacy, it has been shown that results obtained with rodent prions cannot be fully extrapolated to inactivation of human prions (22, 26).

Recently, *in vitro* amplification techniques that aim to detect minute amounts of PrP^TSE^ have been developed. Protein misfolding cyclic amplification (PMCA) and real-time quaking-induced conversion (RT-QuIC) rely on the self-propagating property of prions and allow detection of the conversion of PrP^C^ into amyloid aggregates (seeding activity) (27, 28). Such approaches are very promising for the diagnosis of CJD (29–31) and for the first time allowed the detection of variant CJD (vCJD) prions in the plasma of two individuals more than 2 years before clinical onset of disease (7).

In a previous study, by using PMCA associated with contaminated steel wires (Surf-PMCA), we were able to show that the sensitivity of the Surf-PMCA method allows discrimination of decontamination treatments with respect to their effectiveness on vCJD prions by monitoring residual seeding activity (RSA) on wires (32). However, the results obtained with prion-specific commercial solution showed variable effectiveness to alter the seeding activity of the vCJD prion. Even if compelling evidence supports a relationship between seeding activity and infectivity (33–37), here we complete our previous *in vitro* results with *in vivo* transmission studies. We used vCJD susceptible mice implanted with steel wires that were contaminated by vCJD prions and subjected to different decontamination procedures to demonstrate a good concordance between RSA measured by Surf-PMCA and residual infectivity.

## RESULTS

### Endpoint titration of vCJD infectivity bound to steel wires.

Using steel wire contaminated with serial dilutions of vCJD infectious brain homogenate (vCJD-IBH), we performed an endpoint titration to compare *in vitro* PMCA results with the tgBov (transgenic mice overexpressing the physiological level of bovine PrP by 6 times) bioassay. This mouse model was used because of its capacity to succumb to low doses of vCJD prions (38). Steel wires were contaminated individually with serial dilutions of vCJD-IBH, from 10^−1^ to 10^−8^, or 10^−1^ human normal brain homogenate (hu-NBH) as a negative control. Wires were then implanted intracerebrally into tgBov mice and observed for up to 700 days postimplantation (d.p.imp). Results are summarized in Table 1. Each group started with 10 mice, but some challenged animals died from intercurrent disease, possibly owing to the invasiveness of the procedure. Accordingly, the number of animals in the respective groups was readjusted. Animals implanted with 10^−1^ or 10^−2^ dilution vCJD-contaminated steel wires showed an attack rate of 90% and 89%, respectively, with one mouse lost for intercurrent reasons in the 10^−2^ group. Survival times were similar with 406 ± 9 and 413 ± 31 days, respectively. For animals implanted with 10^−3^ and 10^−4^ dilution vCJD-contaminated steel wires, the attack rate was 50%, with two mice lost in the 10^−3^ group. Survival times were again equivalent with 532 ± 42 and 515 ± 43 days, respectively. Finally, 1 out of 10 mice from the 10^−5^ dilution vCJD-contaminated steel wire group succumbed to the disease in 462 days and allowed us to determine the dilution limit. This makes it possible to study prion infectivity reduction dynamics over 4 log_10_ units. None of the subsequent groups (10^−6^, 10^−7^, 10^−8^, and negative [Neg]) died of TSE. At the 10^−5^ dilution limit, the transmission rates in the adsorbed prion group with implanted wires and nonadsorbed prion group (positive control) were quite similar, suggesting a relatively limited loss of infectivity due to binding.

**TABLE 1:** Endpoint titration of infectivity of vCJD bound to steel wires

Transgenic line	Inoculum^*a*^	Attack rate (no. of positive mice/total no. of mice)	Transmission rate (%)	Survival time (no. of days) (mean ± SEM)
tgBov	10^−1^ SW	9/10	90	406 ± 9
	10^−2^ SW	8/9	89	413 ± 31
	10^−3^ SW	4/8	50	532 ± 42
	10^−4^ SW	5/10	50	515 ± 43
	10^−5^ SW	1/10	10	462
	10^−6^ SW	0/10	0	>700
	10^−7^ SW	0/10	0	>700
	10^−8^ SW	0/10	0	>700
	hu-NBH SW	0/10	0	>700
	10^−3^ IBH	10/10	100	342 ± 16
	10^−5^ IBH	1/9	11	567

tgHu	10^−2^ SW	11/11	100	776 ± 22
	10^−3^ SW	6/6	100	723 ± 48
	Not inoculated	0/5	0	>700

a SW, steel wire; hu-NBH, normal brain homogenate (NIBSC NBHZO/0005); IBH, infected-brain homogenate (vCJD NIBSC NHBYO/0003).

This suggests that the model of vCJD prions adsorbed on steel wires combined with the bovine PrP mouse is a relevant model to validate decontamination formulation against vCJD prions.

In parallel, 10^−2^ and 10^−3^ dilution vCJD-contaminated steel wires were implanted in tgHu (transgenic mice overexpressing the physiological level of human PrP by 6 times) mice (tg650 line) or otherwise used as the substrate for vCJD amplification by Surf-PMCA. Indeed, all mice died of TSE (11/11 and 6/6, respectively) but with a survival time exceeding 700 d.p.imp, suggesting a limited dynamic range for the evaluation of vCJD decontamination procedures compared with tgBov mice.

### Evaluation of standard and commercial decontamination procedures on vCJD-contaminated steel wires *in vivo*.

**FIG 1:**
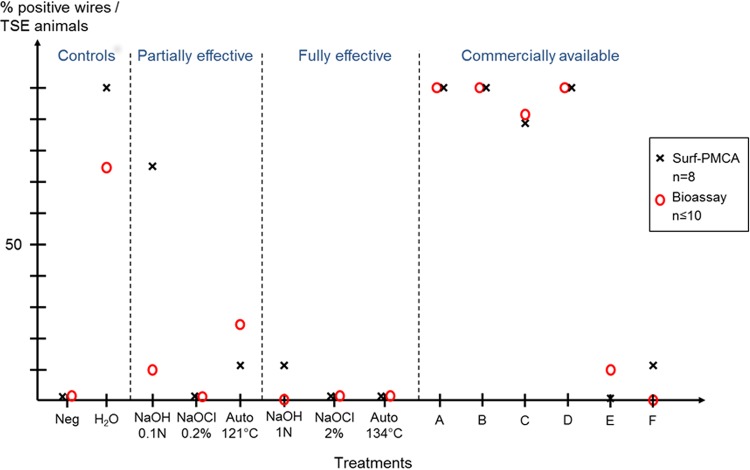
Evaluation of standard and commercially available decontamination procedures on vCJD prions. Steel wires contaminated with 10% human vCJD brain homogenate were treated by standard and commercially available prion decontamination procedures and subjected to four serial rounds of PMCA (Surf-PMCA) (black crosses) (results from Belondrade et al. [32]) or inoculated in tgBov mice (Bioassay) (red circles). Treatments are as follows: Neg, negative control (wire mock contaminated with normal brain homogenate); H_2_O, untreated ineffective treatment control; NaOH, sodium hydroxide (0.1 N for 15 min or 1 N for 60 min); NaOCl, sodium hypochlorite (0.2% [2,000 ppm] for 15 min or 2% [20,000 ppm] for 60 min); Auto, steam sterilization at 121°C for 20 min or at 134°C for 20 min.

We then compared our previous *in vitro* data obtained by Surf-PMCA with infectivity using the tgBov transmission model. vCJD-contaminated steel wires (10^−1^ dilution) treated by standard and commercial decontamination treatments were implanted in the brains of tgBov mice, and the mice were monitored for TSE development (Table 2). Standard prion decontamination methods (i.e., 20,000 ppm sodium hypochlorite, 1 mol/liter sodium hydroxide, or steam sterilization at 134°C), as well as milder counterpart methods (i.e., 2,000 ppm sodium hypochlorite, 0.1 mol/liter sodium hydroxide, or steam sterilization at 121°C), were used as fully and partially effective controls, in addition to water as an ineffective control. The fully effective standard treatments led to 100% survival of the animals; in addition, the substandard 2,000 ppm sodium hypochlorite treatment had a 100% survival rate, confirming a good efficacy of these treatments on vCJD prions. Analysis of the brains of animals at the time of euthanasia showed the absence of PrP^TSE^, thus confirming the absence of infection. Regarding the remaining partially effective treatments, 1 mouse out of 10 developed TSE in the 0.1 mol/liter sodium hydroxide-treated group (transmission rate, 10%), and 2 mice out of 8 died with disease in the group inoculated with steel wires sterilized at 121°C (transmission rate, 25%). These results are concordant with *in vitro* data obtained by the monitoring of RSA by Surf-PMCA (Fig. 1). Surprisingly, the water-only control group had a transmission rate of 75%, whereas 100% was expected. In the asymptomatic mice from this group, steel wires were recovered from their brains after autopsy, meaning there was no lack of implantation. Moreover, we performed PMCA amplification in the brains and could not detect PrP^TSE^, confirming the uninfected status of these two mice (data not shown). Unfortunately, we lost two mice of this group from intercurrent disease very early postinoculation, and the number of mice in this group was reduced to eight, which could be important with regard to the transmission rate of 75%. However, results obtained for the six other mice of this group are very consistent with 10^−1^ dilution steel wire (SW) group with regard to the incubation period.

**TABLE 2:** Evaluation of standard and commercially available decontamination procedures on vCJD-contaminated steel wires by tgBov

Classification and decontamination procedure	Infectivity study			Surf-PMCA^*a*^	
	Attack rate (no. of positive mice/total no. of mice)	Transmission rate (%)	Survival time (no. of days) (mean ± SEM)	RSA detection (no. of positive wires/total no. of wires)	PMCA rounds needed for PrP^TSE^ detection
Ineffective treatment					
Water - 60 min	6/8	75	428 ± 14	8/8	Rd 1

Partially effective treatments					
Sodium hydroxide 0.1 N - 15 min	1/10	10	433	6/8	Rd 3/4
Sodium hypochlorite 0.2% - 15 min	0/10	0	>700	0/8	
Steam sterilization 121°C - 20 min	2/8	25	376, 475	1/8	Rd 4

Fully effective treatments					
Sodium hydroxide 1 N - 60 min	0/8	0	>700	1/8	Rd 4
Sodium hypochlorite 2% - 60 min	0/8	0	>700	0/8	
Steam sterilization 134°C - 20 min	0/7	0	>700	0/8	

Anonymized marketed treatments^*b*^					
A	10/10	100	376 ± 13	8/8	Rd 1
B	8/8	100	490 ± 19	8/8	Rd 2/3
C	9/10	90	451 ± 17	7/8	Rd 2/3
D	10/10	100	431 ± 18	8/8	Rd 2/3
E	1/10	10	460	0/8	
F	0/10	0	>700	1/8	Rd 3

a Results are from Belondrade et al. (32). RSA, residual seeding activity; Rd, round of PMCA.

b Marketed treatments have been approved by the French regulatory agency (ANSM).

More interestingly, of the six commercial treatments evaluated using the tgBov bioassay, only one displayed total vCJD prion decontamination. Anonymized treatments A, B, C, and D had transmission rates of 100, 100, 90, and 100%, respectively, with survival time slightly higher than the water-only control group (Table 2). Treatment A seemed to slightly potentiate vCJD infectivity considering the lower survival time compared to the water-only group (376 ± 13 and 428 ± 14, respectively). PrP^TSE^ was detected on all wires after only one round of Surf-PMCA (Table 2 and Fig. 1). Treatments E and F gave the best results with transmission rates of 10% and 0%, respectively, which were very close to the fully effective standard treatments. Again, *in vivo* data are concordant with RSA determined by Surf-PMCA, which gave also maximal efficacy for treatments E and F with 0% and 10% of vCJD-positive wires detected, respectively (Fig. 1).

### Surf-PMCA regenerates infectivity.

**FIG 2:**
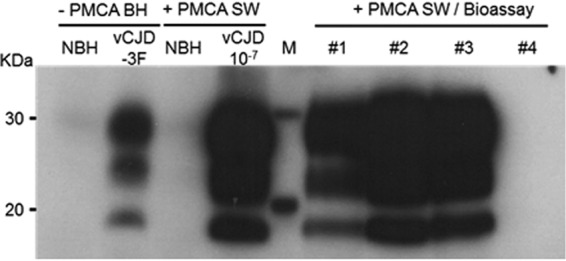
Surf-PMCA regenerates infectivity. Western blot analysis of protease-resistant prion protein from original brain homogenates (− PMCA BH), Surf-PMCA amplicons (+ PMCA SW), and brain extract from mice inoculated with the third round of Surf-PMCA amplicons (+ PMCA SW/Bioassay) either from non-CJD or vCJD patient. Prediluted samples (20-μl samples) were digested with proteinase K before Western blot analysis. The two leftmost lanes (− PMCA BH lanes) contain normal (NIBSC NBHZO/0005) (10%) and vCJD (NIBSC NHBYO/0003) (0.1%) brain homogenate (wt/vol) used for initial steel wire contaminations. The + PMCA SW lanes contain Surf-PMCA amplicons (third round) from steel wire exposed to NBH or 10^−7^ vCJD dilution. The + PMCA SW/Bioassay lanes contain brain extract from mice inoculated with the third round of Surf-PMCA amplicons obtained with 10^−7^ dilution vCJD-contaminated steel wires (lanes #1 to #3) or with hu-NBH steel wire (lane #4). Molecular masses (in kilodaltons [KDa]) are indicated in lane M. Protease-resistant prion protein was detected with 9A2 monoclonal antibody.

In order to assess the capacity of Surf-PMCA to regenerate vCJD infectivity, we inoculated PMCA amplicons from 10^−7^ dilution vCJD-IBH- or hu-NBH-contaminated steel wires to tgBov mice (Table 3). Because the Triton X-100 (1%) contained in the converting buffer used in the PMCA reaction is toxic to intracerebrally inoculated animals, we had to dilute the amplicons 10 times in phosphate-buffered saline (PBS). In parallel, 10^−3^ and 10^−5^ dilutions of vCJD-IBH were also inoculated as previously mentioned (Table 1). Figure 2 illustrates the protease-resistant prion protein observed from the initial human brain materials, the generated Surf-PMCA amplicons obtained from wires contaminated with those brains or from brain extracts of mice inoculated with the generated amplicons (amplicons 1 to 4). Western blot profiles indicate a conservation of the vCJD molecular signature among the positive samples with a typical type 2 unglycosylated band at 19 kDa and a predominance of the diglycosylated isoform. Mice inoculated with PMCA amplicons from 10^−7^ dilution vCJD-contaminated steel wires had a transmission rate of 100% with a survival time of 338 ± 4 days postinoculation (d.p.inoc) (Table 3). Animals inoculated with negative PMCA amplicon (from hu-NBH-steel wires) had a transmission rate of 0%. Interestingly, all mice inoculated with 10^−3^ dilution of vCJD-IBH succumbed to TSE in 342 ± 16 d.p.inoc, a survival time similar to amplicon-inoculated mice.

**TABLE 3:** Infectivity study of Surf-PMCA amplicons in tgBov

Surf-PMCA- amplified material^*a*^	Attack rate (no. of positive mice/total no. of mice)	Transmission rate (%)	Survival time (no. of days) (mean ± SEM)
10^−7^ SW	9/9	100	338 ± 4
hu-NBH SW	0/10	0	>700

a hu-NBH, human normal brain homogenate (NIBSC NBHZO/0005); SW, steel wire.

Considering the small amount of infectious material adsorbed on a single steel wire and the 1-log-unit dilution factor before inoculation, we showed that three rounds of Surf-PMCA could regenerate 4 log units of infectivity.

## DISCUSSION

We demonstrated in this study that our previous results obtained by monitoring the RSA by Surf-PMCA for the evaluation of decontamination procedures against vCJD prions were concordant with the measure of residual infectivity in tgBov mice.

The choice of the transgenic model for the *in vivo* assay was driven by literature review. Although vCJD PrP^TSE^ and bovine PrP^C^ differ with regard to their primary sequence, the capacity of vCJD prions to transmit in transgenic mice overexpressing bovine PrP without species barrier has been comprehensively described (39). Due to the absence of this species barrier, it has been reported previously that tgBov line 110 mice were highly susceptible to vCJD prions (38, 40–42). vCJD represents human infection with bovine spongiform encephalopathy (BSE) from cattle. This phenomenon has been designated “traceback,” and traceback studies have been proven to be a useful tool to identify the origin of prions (39, 43, 44). These results suggest that BSE prions retain their host preference after repeated passages through human PrP (42), as in other species, including sheep, cat, and mouse. It must also be noted that bovine PrP mice succumb quicker with vCJD prions but are intrinsically not more susceptible than tgHu line 650 mice to vCJD prions (45). Although homogenate substrate used for Surf-PMCA originated from tgHu mice (overexpressing 6 times the physiological level of human PrP) (46), survival time bioassays based on this transgenic line are limited by the long incubation period of vCJD (exceeding 500 d.p.inoc when undiluted) (45). tgBov mice (overexpressing 6 times the physiological level of bovine PrP) (47) were previously used and showed a good dynamic range with one animal dying with up to a 10^−6^ dilution of a vCJD brain homogenate in 500 d.p.inoc (38). Our results are concordant with these data, as 10^−2^ and 10^−3^ dilution vCJD-contaminated steel wires implanted in tgHu mice gave an attack rate of 100%; however, the incubation time exceeded 700 d.p.imp—almost the life span of the animals—which precludes their use to monitor a reduction factor. On the other hand, tgBov mice showed a better dynamic range with one animal dying after exposure with a 10^−5^ dilution vCJD-contaminated steel wires. Surprisingly, whereas attack rates and incubation times obtained with the 10^−3^ vCJD dilution indicated an expected lower infectivity of steel wire-bound prions than with the diluted prions, very similar results were obtained at the 10^−5^ dilution limit for the two groups (wires or dilution). One explanation could be that in the case of very low quantity of prions, the process used for wire contamination with IBH (one single wire per well and air drying overnight) may have potentiated vCJD transmission. A second hypothesis would rely on the longer total brain local exposure of mice with wires that remain in the brain for a long time, in contrast to injected brain homogenates that circulate immediately. Despite the relevance of the tgBov model as a vCJD bioassay, the limited dynamic of steel wire endpoint titration (50% attack rate for animals implanted with 10^−4^ dilution vCJD-contaminated steel wires) and its nonlinear decrease did not allow the calculation of an accurate 50% endpoint titer. Furthermore, although there were initially 10 mice per implantation condition, some animals were lost, which limited statistical significance. However, compared with the 263K hamster model, in which the dilution limit is 10^−6^ diluted contaminated steel wires (22% transmission rate) (19), the 10^−5^ dilution limit we obtained with tgBov inoculated with vCJD prions is only 1 log unit less sensitive and allows the comparison of the residual infectivity with the RSA measured by Surf-PMCA. Therefore, when vCJD-contaminated steel wires were treated by either standard or commercial treatments, the tgBov model was sensitive enough for results to be interpreted. Fully effective standard treatments showed no transmission of vCJD in the tgBov mice model. Except for sodium hypochlorite at 2,000 ppm, the other partially effective treatments led to few animals developing TSE. As inferred from the Surf-PMCA results, out of six commercial treatments, four poorly decreased the infectious load adsorbed on steel wires, with treatment A seeming to shorten the survival time compared with the water-only control. Despite PMCA being demonstrated as more sensitive than bioassays by several log units of magnitude for the detection of prions (34, 35, 48, 49), and that, to our knowledge, this is the first time the steel wire assay has been used with vCJD prions, we showed a high concordance between the Surf-PMCA results and the use of steel wires as vCJD carrier in transgenic mice.

We demonstrated using Surf-PMCA and tgBov infectivity studies that some of the commercial chemicals tested were not fully effective for decontaminating vCJD prions on surfaces. However, all these treatments were approved regarding their efficacy on the 263K prion strain. Our results confirm the inaccuracy of 263K prions regarding the validation of decontamination procedures used in health care facilities for the inactivation of vCJD prions. Nevertheless, our results regarding the effectiveness of vCJD prion decontamination by marketed reagents should be mitigated owing to the specific experimental set up whereby wires were air dried after contamination. Although all reagents evaluated in this study were previously validated using similar prion-dried conditions (using 263K prion-contaminated steel wires in hamsters), it is important to note that in health care facilities, it is recommended that MDs are kept continuously moist before prion decontamination. Whether vCJD-bound prions would behave differently if steel wires are kept moist after contamination remains to be established. Our Surf-PMCA method should be able to provide complementary data to help manufacturers of products to evaluate and improve their effectiveness in more real conditions. In addition, although TSE agents have notable extreme resistance to most decontamination processes, iatrogenic transmission of CJD via neurosurgical instruments has been reported in only four cases worldwide, and two cases have occurred because of contaminated stereotactic electroencephalography (EEG) depth electrodes in Switzerland (12). No new cases of iatrogenic transmission of CJD have been reported for several decades, underlining the poor transmission efficiency and the probable effectiveness of risk management procedures currently in place in health care facilities.

Recently, vCJD diagnosis has been possible in plasma samples from clinical and preclinical patients using the PMCA amplification technique (7) and in cerebrospinal fluid samples from clinical vCJD patients, including the first heterozygous methionine/valine patient (29), who might be the first case of a feared second wave of vCJD cases (50, 51). The capacity of PMCA to regenerate infectivity has already been demonstrated on nonhuman prions such as with scrapie prions, for which Moudjou et al. showed that infectivity of a 10^−9^ dilution of infected brain amplified by one round of PMCA was similar to that of the initial brain (34). Although sometimes debated (52, 53), the infectivity of vCJD PMCA amplicons, as well as the capacities of PMCA amplicons to faithfully maintain the pathological features of the original inoculum, led us to challenge tgBov mice with PMCA amplicons obtained from 10^−7^ vCJD-contaminated steel wires. By comparing the results with those obtained with a 10^−3^ vCJD-IBH dilution, we observed an equivalent survival time, demonstrating the ability of PMCA to regenerate at least 4 log units of vCJD infectivity. We also obtained a similar profile on Western blots for the PrP^TSE^ present in the mouse brains. Altogether, these results confirmed that an RSA detected by the Surf-PMCA assay can be linked to residual infectivity in mice.

To extend the use of Surf-PMCA for the evaluation of prion decontamination treatments, it could be of interest to adapt it to other human prions, in particular sporadic CJD prions which represent 85% of TSE cases. Considering the differences of the biochemical properties of PrP^TSE^ among the different sporadic CJD (sCJD) subtypes, such as solubility in detergents, heat stability, or sensitivity to protease digestion (54–56), the behavior of non-vCJD human prions should be considered with regard to decontamination procedures. Similarly, other protein misfolding diseases such as Alzheimer’s and Parkinson diseases should also be considered on a precautionary basis in the development of decontamination procedures adapted to MDs (57). Adaptation and automation of Surf-PMCA would be of significant interest for a rapid and low-cost evaluation of new decontamination processes regarding misfolding diseases.

## MATERIALS AND METHODS

### Infectious material.

vCJD infectious brain homogenate (vCJD-IBH) and human normal brain homogenate (hu-NBH) were provided by the UK National Institute for Biologicals and Standards (CJD resource center at https://www.nibsc.org/) as 10% (wt/vol) homogenates in 0.25 mol/liter sucrose (reference WHO NHBYO/0003 and NBHZO/0005).

### Preparation and contamination of stainless steel wires.

Stainless steel wires (catalog no. FE245102; Goodfellow, England) (diameter, 0.15 mm) were prepared as previously described (32). Briefly, batches of wires were cut into 3-mm pieces and contaminated in 96-well plates (one wire per well) with serial dilutions of vCJD-IBH (10^−1^ to 10^−8^) in order to evaluate bioassay sensitivity (Table 1). After air drying, wires were rinsed with phosphate-buffered saline (PBS) and individually stored at –80°C prior to implantation in animals.

For decontamination evaluation or negative controls, wires were incubated in 10% vCJD-IBH or 10% hu-NBH, respectively. After air drying, wires were individually stored at –80°C prior being processed.

### Processing of stainless steel wires.

Before processing, 96-well plates containing contaminated wires were dried at room temperature. Wires were then exposed to the different formulations or procedures for prion disinfection listed in Table 2 and Table S1 in the supplemental material as previously described (32). Briefly, test wires were incubated in 200 μl of the different treatment solutions and handled according to the manufacturer’s instructions. For steam sterilization, wires were placed in a ceramic plate. After treatment, wires were individually stored at –80°C prior to implantation in animals.

10.1128/mSphere.00649-19.1 TABLE S1 List of prion-inactivating reagents used in this study and operating conditions. Download Table S1, PDF file, 0.01 MB. Copyright © 2020 Bélondrade et al. 2020 Bélondrade et al. This content is distributed under the terms of the Creative Commons Attribution 4.0 International license.

### Surf-PMCA.

Surf-PMCA was performed as previously described (32). Briefly, wires contaminated with 10^−7^ dilution of vCJD-IBH or the negative PMCA control, hu-NBH, were serially amplified using normal brain homogenate (NBH) from humanized transgenic mice (M129 allele, tg650 line) as the substrate for PMCA. One PMCA round was composed of 80 cycles of 20 s of sonication at 220 to 240 W power followed by 29 min 40 s of incubation at 37°C in a Q700 microplate horn sonicator (Qsonica). Ten microliters of amplified product was mixed with 90 μl of fresh NBH and subjected to an additional PMCA round of 80 cycles. Three rounds of Surf-PMCA were performed in this study.

### Animal transmission studies.

These animal experiments, performed in biosafety level 3 laboratories, were authorized by the Institut National de la Recherche Agronomique (INRA) ethics committee (approval number 12-034).

Female transgenic mice overexpressing 6 times the physiological level of bovine PrP (tg110 line [tgBov]) (47) were intracerebrally inoculated with one steel wire prepared as described above. Mice were anesthetized intraperitoneally with a mixture of ketamine and xylazine before being implanted. Local anesthesia with lidocaine was applied, and the mice were kept on a heating mat at 37°C until waking.

Different groups of 10 mice distributed in two cages (Tables 1 and 2) were inoculated as follows. Negative controls were represented by wires incubated in hu-NBH or uncoated/unprocessed wires, positive controls were intracerebrally inoculated with 20 μl of 10^−3^ and 10^−5^ dilutions of vCJD-IBH (used for steel wire contamination). Endpoint titration was determined with wires contaminated with serial dilutions of vCJD-IBH from 10^−1^ to 10^−8^, and decontamination procedures were assessed using wires contaminated with 10^−1^ vCJD-IBH and processed according to the different procedures.

Furthermore, in order to compare the sensitivity of humanized mice, which overexpress by 6 times the physiological level of Met129 human PrP [tg650 line (tgHu)] (46), with the tgBov model, 10^−2^ and 10^−3^ vCJD- contaminated wires were also intracerebrally implanted in two groups of 6 to 12 mice.

Finally, we compared the infectivity of a PMCA amplicon obtained after three Surf-PMCA rounds of a 10^−7^-contaminated wire with nonamplified vCJD-IBH by intracerebrally inoculating 20 μl of the amplicon diluted 10 times in PBS in tgBov mice. A negative PMCA amplicon obtained after three Surf-PMCA rounds of a wire coated with hu-NBH was inoculated in parallel.

Mice were monitored daily for clinical sign of TSE. Animals at the terminal stage of the disease or with intercurrent pathology or after asymptomatic survival for an observation period of >700 days postimplantation (d.p.imp) or postinoculation (d.p.inoc) were euthanized by cervical elongation or lethal injection of pentobarbital. Survival times and attack rates for the development of terminal TSE were monitored, and diagnosis of TSE was confirmed by detection of PrP^TSE^ in brains by Western blotting using biotinylated Sha31 antibody (58).

### Proteinase K digestion and SDS-PAGE/immunoblotting.

A volume of 20 μl of each sample, prediluted or not, was incubated at 45°C with proteinase K (200 μg/ml) for 1 h before denaturation at 100°C in sodium dodecyl sulfate-polyacrylamide gel electrophoresis (SDS-PAGE) sample buffer. Samples were run on 12% NUPAGE gels and electrotransferred onto polyvinylidene difluoride (PVDF) membranes. Western blots (using the SNAP ID system [Millipore]) were performed using 9A2 anti-PrP monoclonal antibodies (Wageningen, Netherlands), and an anti-mouse IgG peroxidase-linked secondary antibody (GE Healthcare, UK) linked to a chemiluminescent reaction (ECL blotting detection reagent, GE Healthcare Life Sciences, Amersham, France). For the confirmation of TSE diagnosis in mice, PrP^TSE^ was detected following the protocol described previously using Sha31 antibody.
