# Supplementary material for: Correlation between Bioassay and Protein Misfolding Cyclic Amplification for Variant Creutzfeldt-Jakob Disease Decontamination Studies
Source: mSphere. 2020 Jan 29;5(1):e00649-19. doi: 10.1128/mSphere.00649-19 (PMC6992370; doi:10.1128/mSphere.00649-19)
Supplement: TABLE S1 [file mSphere.00649-19-st001.pdf]

Supplemental Material

S1 Table. List of prion inactivating reagents used in this study and operating conditions.

**Product Name** ***(corresponding letter)***	**Manufacturer**	**Operating conditions**
**ACTANIOS HLD *(C)***	ANIOS	Immersion / ready to use / RT / 30 min
**ACTANIOS HLD**<br>**+ ACTANIOS P1 *(D)***<br>**+ACTANIOS P2**	ANIOS	-P1: immersion/ 0.5% / RT / 10 min<br>-Rinsing<br>-P2: immersion/ 0.5% / RT / 5 min<br>- Rinsing<br>-HLD: immersion / ready to use / RT / 15 min
**ACTANIOS LDI *(E)***	ANIOS	Washer-disinfectors, sterilizing tunnel / 1% / 55°C / 10 min
**ALKA 100 *(A)***	ALKAPHARM	Immersion / 1% RT / 15 min
**HAMO 100 *(B)***	STERIS	Immersion, Washer-disinfectors, sterilizing tunnel / 0.8% / 43°C / 7.5 min
**NEODISHER SEPTOCLEAN *(F)***	DR WEIGERT	-Immersion / 1% / RT / 60 min<br>-Washer-disinfectors, sterilizing tunnel / 1% / 55°C / 10 min

Reagents have been validated by the French regulation Agency (ANSM) (version of 4/4/2012)

RT: Room temperature
